# A microcarrier-based spheroid 3D invasion assay to monitor dynamic cell movement in extracellular matrix

**DOI:** 10.1186/s12575-019-0114-0

**Published:** 2020-02-01

**Authors:** Hui Liu, Tao Lu, Gert-Jan Kremers, Ann L. B. Seynhaeve, Timo L. M. ten Hagen

**Affiliations:** 1 Laboratory of Experimental Oncology, Department of Pathology, Erasmus Medical Center, Rotterdam, the Netherlands; 2 Erasmus Optical Imaging Center, Erasmus Medical Center, Rotterdam, the Netherlands

**Keywords:** Cell invasion, 3D, Microcarrier beads, Spheroids, Time-lapse microscopy, Quantification

## Abstract

**Background:**

Cell invasion through extracellular matrix (ECM) is a critical step in tumor metastasis. To study cell invasion in vitro, the internal microenvironment can be simulated via the application of 3D models.

**Results:**

This study presents a method for 3D invasion examination using microcarrier-based spheroids. Cell invasiveness can be evaluated by quantifying cell dispersion in matrices or tracking cell movement through time-lapse imaging. It allows measuring of cell invasion and monitoring of dynamic cell behavior in three dimensions. Here we show different invasive capacities of several cell types using this method. The content and concentration of matrices can influence cell invasion, which should be optimized before large scale experiments. We also introduce further analysis methods of this 3D invasion assay, including manual measurements and homemade semi-automatic quantification. Finally, our results indicate that the position of spheroids in a matrix has a strong impact on cell moving paths, which may be easily overlooked by researchers and may generate false invasion results.

**Conclusions:**

In all, the microcarrier-based spheroids 3D model allows exploration of adherent cell invasion in a fast and highly reproducible way, and provides informative results on dynamic cell behavior in vitro.

## Background

Malignant tumors have the potential to metastasize from the original tissue to a distant site, which is the main cause of morbidity and mortality in tumor patients. During this process, the basic but decisive step is migration of tumor cells through the extracellular matrix (ECM) either towards lymph and blood vessels, or to a secondary site after survival in circulation [1]. To disseminate in tissue, cells require adhesion, proteolysis of ECM components and migration, which also occurs in normal physiological processes like embryonic morphogenesis and wound healing [2]. There are a diversity of strategies for cells movement, either individually (e.g. amoeboid or mesenchymal migration) or collectively (multicellular streaming, cluster, strand or sheet), which are based on cell-cell adhesion and cell-matrix interaction [3–5]. This activity can be simulated and observed by in vitro models and optical imaging to study cellular and molecular mechanisms. Unlike 2D migration, a 3D matrix provides both a substructure and obstacles to all surfaces of cells during movement through the surroundings, which simulates cell movement through tissues. Importantly, 3D models provide more information on the process of cell migration and invasion, including cell morphological alterations, cell-cell interaction, cell-matrix interaction, and matrix remodeling. Therefore, 3D models can serve as a supplement or an advanced alternative to 2D assays.

To examine cell invasive potential, a variety of in vitro assays are developed in a 3D format. Among them the Transwell invasion assay, or Boyden chamber assay, is widely used. Basically it includes seeding cells on a layer of ECM gel pre-coated on top of a porous membrane, and assessing cell invasion by measuring the number of cells passing through the ECM gel. The chamber invasion assay is straightforward to quantify invading cells induced by chemoattractants [6] or internal gene regulation [7]. Despite the advantages, this assay counts vertically invading cell numbers at the endpoint but neglects the whole invasion process. How cells move in matrix and interact with surroundings remains unclear. As a substitute, a matrix embedding cell culture offers more possibilities. Cell aggregates, such as multicellular spheroids, can be embedded in a 3D matrix and cells moving away from spheroids into the matrix are monitored by microscopy. This approach allows cells migrating in any direction and many migratory parameters can be detected, including cell trajectories, migration distance, and velocity. However, establishing spheroids has met with challenges such as absence of formation, lack of size and uniformity control, difficulty in manipulation, requirements of special equipment and training, and is time consuming [8, 9]. Most importantly, not all cells are capable to form tight and regular-shaped spheroids, but some end up as friable and loose aggregates, or aggregation does not occur at all, which complicates manipulation and use in an invasion assay [10–12]. Therefore we choose microcarriers as a core to grow spheroids and to standardize the invasion assay in a simple and highly reproducible way. Adherent cells which do not aggregate spontaneously, can attach to microcarriers and thus form spheroids. Interestingly, introduction of carriers also enables co-culture of different cell types in close proximity [13]. Although microcarrier-based spheroids, because of the core, do not mimic fully the in vivo situation of solid tumors, they are faster to establish and stabilize experimental conditions allowing easy duplication compared to cell-only spheroids. In this study, we describe a microcarrier-based spheroid model to investigate dynamic cell behavior in three dimensional matrices.

## Results

**Fig. 1:**
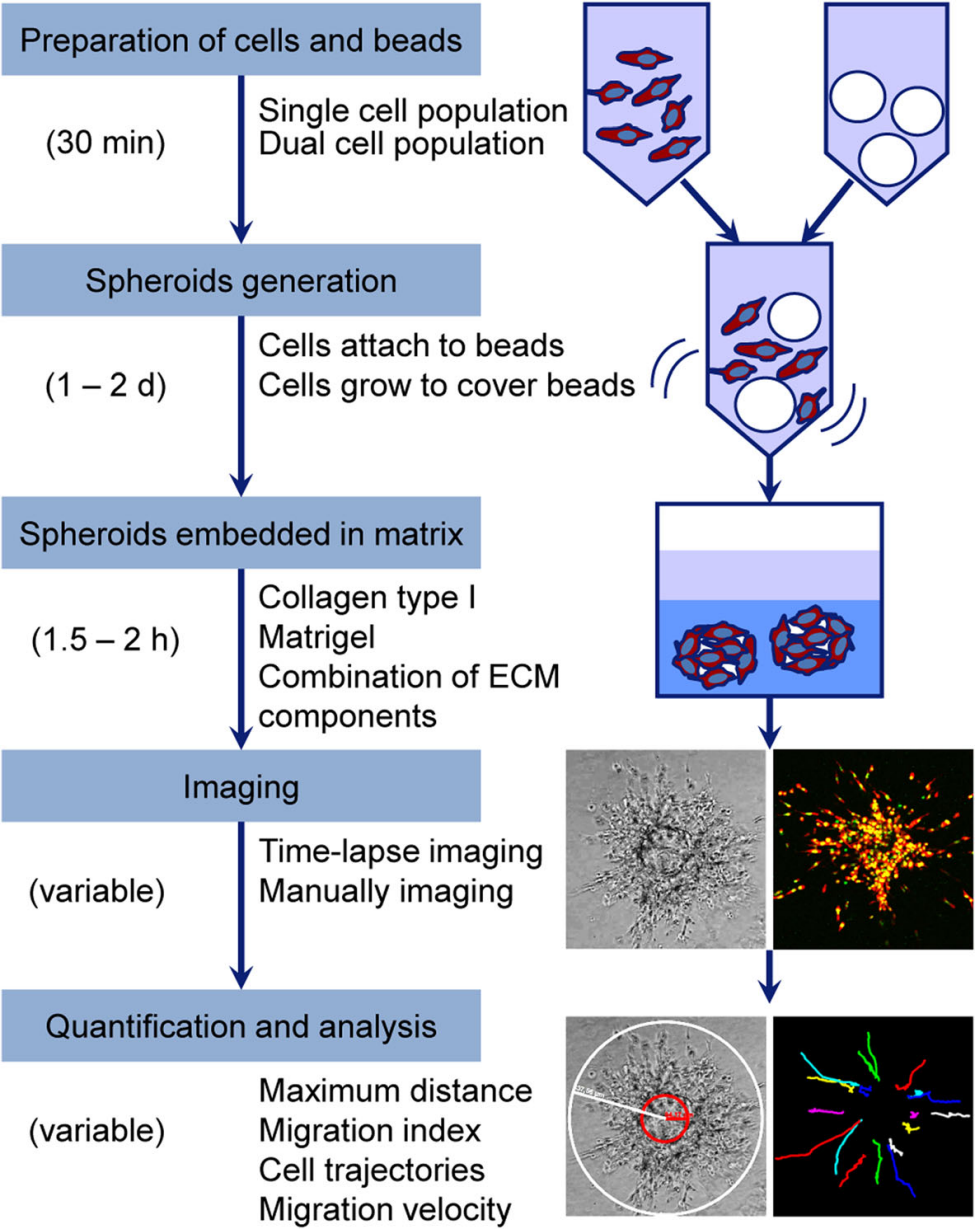
Workflow diagram of the whole assay with schematic drawings and example results

In this study we present a method for 3D invasion examination and introduce various measurements according to different experimental settings and requirements. The whole workflow and schematic diagram is shown in Fig. 1.

### Different Cell Dispersions in Matrix Show Invasiveness

**Fig. 2:**
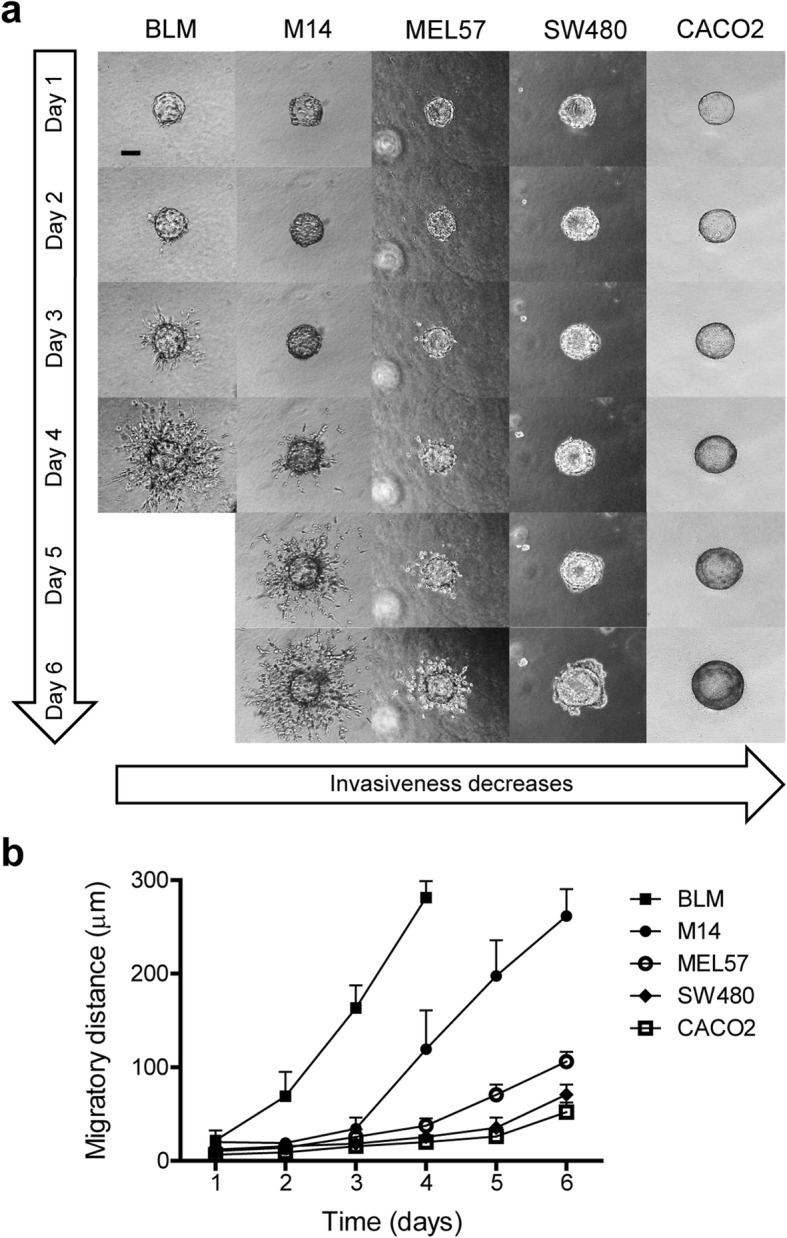
Cell invasion/dispersion in collagen I. Melanoma cells (BLM, M14 and MEL57) and colorectal cancer cells (SW480 and CACO2) were cultured on microcarrier beads and embedded in collagen I gel (1.6 mg/ml). Cell invasion was monitored and recorded daily, and three independent experiments were performed. This assay lasted for 6 days and was ended when cells started to move out of frame. **a** Representative pictures of cell invasion of each cell line. All three melanoma cell lines displayed invasive behavior at different levels, while two colorectal cancer cell lines appeared less invasive, especially CACO2, which showed non-invasive growth. Scale bar, 100 μm. **b** Line graphs show maximum migration distances measured every day of each cell line

This method can be used to monitor invasiveness of adherent cells in vitro. Here we performed the 3D invasion assay with melanoma cell lines (BLM, M14 and MEL57) and colorectal cancer cell lines (SW480 and CACO2) in 1.6 mg/ml collagen I gel. These cell lines were chosen because of difference in cell dispersion in the matrix allowing us show typical invasiveness patterns which may be visible. Images of cell dispersion were obtained every day and the maximum migration distances were measured. Within 4 days BLM cells migrated 285 μm away from the microcarrier core. M14 and MEL57 cells migrated slower than BLM cells, with dispersion of 270 μm and 110 μm in 6 days respectively. All melanoma cells moved collectively in the matrix, but single cells were visible in the front of migrating cells. In comparison, colorectal cancer cells SW480 show less invasive and remained more connected than melanoma cell lines. CACO2 cells grew around the core to multi-layers without any sign of migration into matrices (Fig. 2). The results indicate that this 3D assay can be used to examine cell invasive capacity and the way cells move.

### The Content and Concentration of Matrix Influence Cell Invasion

**Fig. 3:**
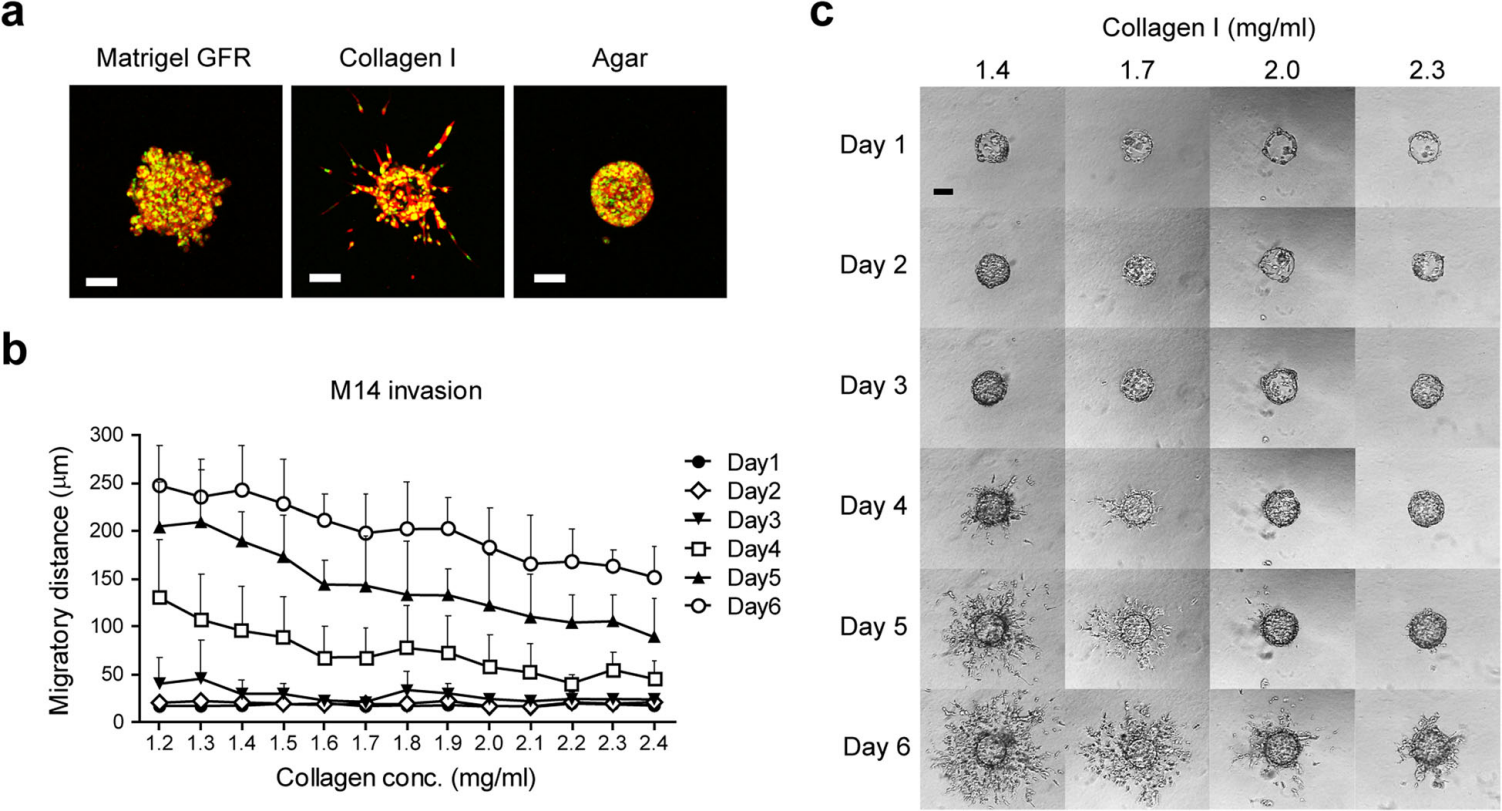
Content and concentration of matrices influence cell invasion. **a** Lewis lung carcinoma (LLC) cells were red fluorescently labeled in cytoplasm and green fluorescently labeled in nucleus. Cell coated microcarrier beads were embedded in 5 mg/ml growth factor reduced (GFR) Matrigel, 1.6 mg/ml collagen I or 0.3% agar respectively, and pictures were taken 56 h later. Scale bars, 100 μm. **b** Melanoma cell line M14 were grown on beads and cell invasion was monitored in a series of concentrations of collagen I gel. Five spheroids were recorded for each individual assay and migratory distance was measured in three independent experiments. Error bars represent standard deviation. **c** Representative pictures of M14 invasion in different concentration of collagen I for 6 days. Scale bar, 100 μm

To investigate the effect of matrix composition on cell invasion, we tried three different types of matrices. Here we use LLC cells because of the individual movement these cells show in collagen. Collagen type I and reconstituted basement membrane (Matrigel) are most commonly used matrices for 3D culture. Agar is a mixture of polysaccharides and can solidify at 32~40 °C for biological use. Fluorescently labeled LLC cells disperse collectively in Matrigel, spread individually in collagen, while no migration was observed in agar (Fig. 3a). Further, to test if the concentration of matrix would influence cell invasion, we used M14 cells in a gradient of collagen matrices and monitored cell invasion in 6 days. We selected M14 cells for the moderate migration speed this cell line shows; not too fast like LLC and BLM, which would move out of the imaging field, or too slow like MEL57, SW480 and CACO2 which demand long culture time causing cell proliferation to affect the migration. The results show a visible descending of migration distances in 4 to 6 days when collagen concentration was increased (Fig. 3b, c). These data demonstrate that different content and concentration of matrix influence cell invasion, so matrix can be adjusted for different experimental design.

### Evaluating the Effect of Treatment on Cell Invasion Using the Migration Index

**Fig. 4:**
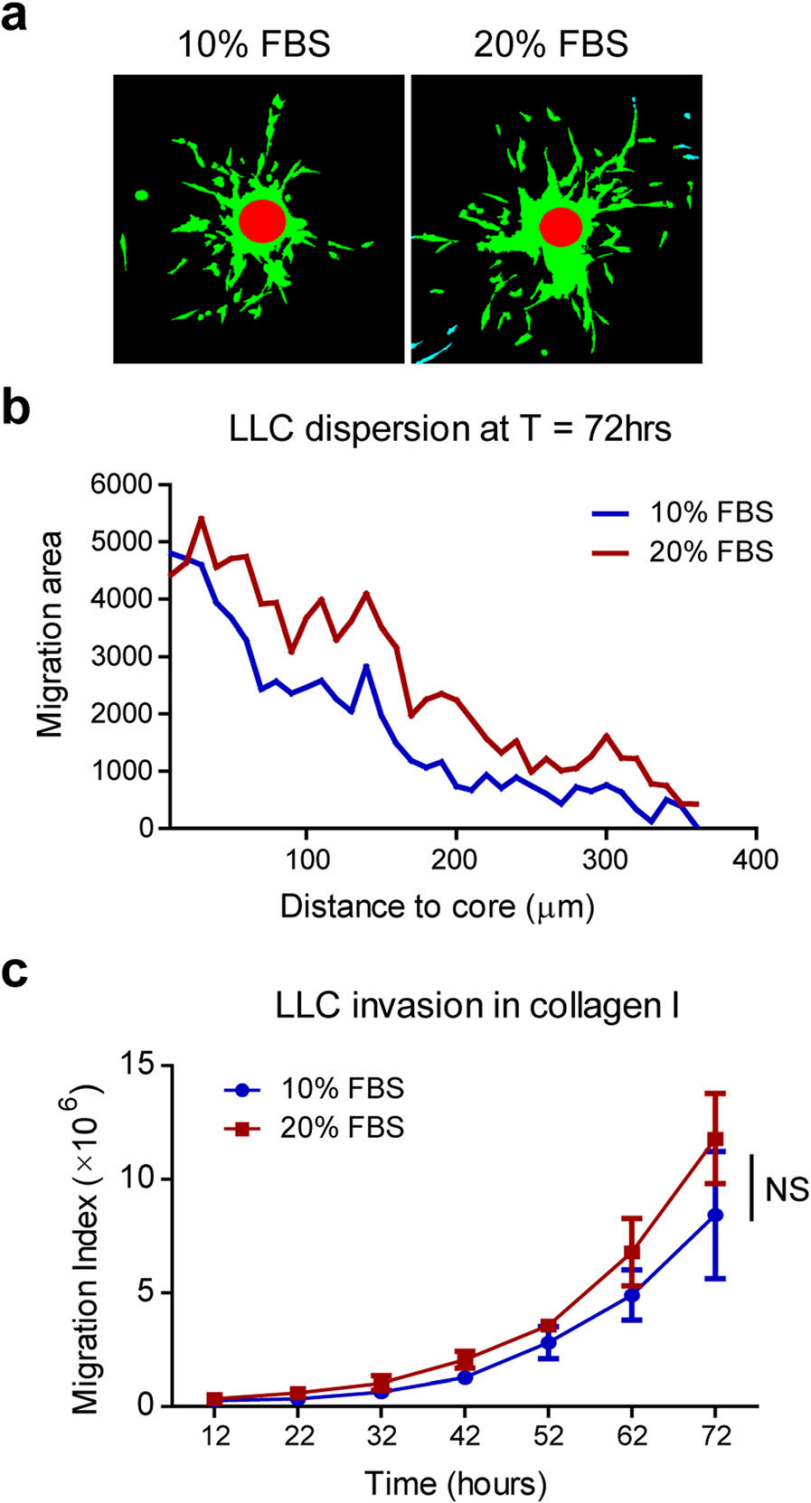
Migration index shows cell invasive capacity. Fluorescently labeled LLC cells were used for invasion test in this 3D assay to compare the effect of 20% FBS vs. 10% FBS. **a** Representative pictures of LLC cell dispersion at 72 h. Cell were color coded for analysis after running additional macros in Fiji. Red circles show microcarrier beads in spheroids, and green areas show distribution of migrating cells at 72 h. **b** Line graph shows migration area changes based on the distance to core at T = 72 h. **c** Calculation of migration index using data of each time point. Data represent mean ± standard deviation (*N* = 3). NS, not significant

To study the effect of a certain treatment on cell invasion, we added extra 10% FBS to a final concentration of 20% in culture medium as treatment, while using DMEM supplemented with 10%FBS as control. To reduce the interference factor of cell division, instead of using collectively migrating cells, we fluorescently labeled LLC cells, which move individually, for confocal time-lapse imaging in three dimensions. Because LLC cells move individually and are scattered in collagen, measuring maximum migration distance, i.e. the distance travelled by one cell furthest from the bead, may exaggerate the real invasiveness and may cause deviation in the data analysis. Therefore we defined a migration index considering the weights of all fast and slowly migrating cells. The migration index is calculated as the sum of all migrating cells multiplied with the distance from the bead. In this setting, fast migrating cells add more values than slowly migrating cells to the migration index, which shows the invasive capacity of the cells together. The cell number is difficult to obtain from images, so cell areas are used to represent cell numbers. Here we used homemade macros (Additional file 1) in Fiji to measure migrating cell areas at every 10 μm away from the core. In Fig. 4a, the red circle shows the microcarrier core and green areas indicate migrating cells included in data analysis. At 72 h, cells with 20% FBS supplemented in medium seem to have larger migration areas at all distance ranges than cells in 10% medium, while the maximum distances in both groups are very close, around 350 μm (Fig. 4b). This result indicates the necessity of introducing the migration index. After computing the migration index of all time points, we found no significant difference between 10 and 20% medium, although an increasing trend was observed in 20% medium (Fig. 4c). The data reveal that the migration index calculation may be affected by increased cell proliferation, and reducing nutrients in the medium will make results of cell invasion more convincing.

### The Position of Spheroids in 3D Matrix Influences Cell Invasion

**Fig. 5:**
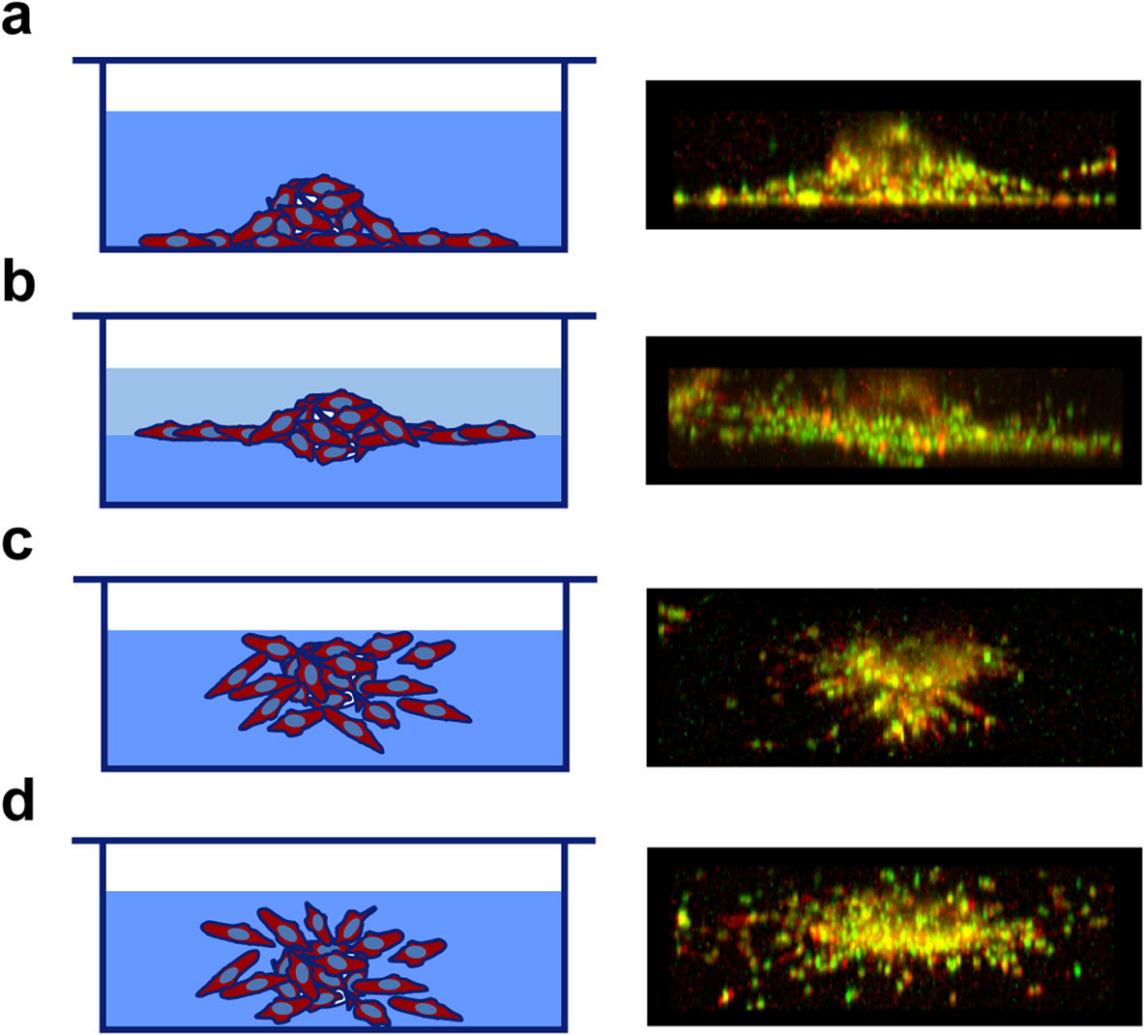
Positions of spheroids in matrices and subsequent cell migration. Schematic diagrams on the left panel indicate corresponding spheroids position of the fluorescent image on the right panel. The images show an x-z view of LLC cells migrating in collagen I. **a** Spheroids sediment at the bottom in matrix and cells tend to follow the interface between gel and bottom surface. **b** A bottom layer of gel was made in the culture plate before adding matrix with spheroids. Most cells move along the interface between the two layers of gel. **c** To prevent spheroids settling, 96-well plate was inverted for 1–2 min at room temperature and spheroids may stay in matrix or near to top. **d** A representative picture of cell dispersing when spheroids are in a proper position of a homogenous collagen I gel

During experiments using this 3D assay, we observed that spheroids might settle at the bottom of the culture plate because of the softness of the gel. When spheroids touch the bottom, most cells prefer migrating along the bottom instead of invading the collagen scaffold (Fig. 5a). This is possibly due to the low resistance in the interface between gel and bottom surface. The spheroids at the bottom cannot be included in data analysis because of exaggerated cell migration distances. If this settlement of beads at the bottom of the well occurs to most spheroids, the matrix concentration might be too low. Normally, increasing the concentration by 0.1~0.2 mg/ml can improve the viscosity of matrix during gel preparation but not reduce migration distance too much (Fig. 3b). In order to avoid beads to settle at the bottom, and to keep the matrix concentration as low as required, we tried to make a sandwich gel consisting of a bottom gel without spheroids and a top gel with spheroids. Interestingly, spheroids could be found in the interface between the two layers of gel and most cells appeared to move in this interface (Fig. 5b). A possible solution could be inverting the culture plate for 1–2 min at room temperature (Fig. 5c), which can, however, only be applied to 96-well format as the well is small enough to retain the viscous liquid. Making use of fluidity of the gel at a certain temperature is another solution. When a low matrix concentration is used, the gel mixed with the cell-coated beads may be pipetted carefully at room temperature to keep the beads in the gel by increasing the viscosity. A proper position of spheroids in the matrix will allow cells to migrate evenly to all directions (Fig. 5d), which shows the innate cell invasion capacity in matrix. Here we show incorrect positions of spheroids in matrices and possible solutions to obtain proper positions for good experiments.

## Discussion

This microcarrier-based spheroid invasion assay provides a powerful approach to assess cell biological behavior in a 3D format, including motility, invasion, angiogenesis, morphological changes, and cell-cell interaction. This method has been used to study the effect of specific gene on cell migration and invasion [14, 15]. It can also be adapted to investigate endothelial cells sprouting and vessel formation [16–18]. After microscopy the gel with invading cells can be fixed for immunofluorescence staining, or can be degraded to isolate cells for further analysis.

The application of microcarrier beads is a fast and highly reproducible way to make spheroids. It allows adherent cells, especially cells which cannot form aggregates with regular shape, to be embedded in matrix as spheroids for invasion study. The microcarrier beads we used in this assay are made of cross-linked dextran coated with a thin layer of denatured collagen. The coating provides a good culture surface for cells to attach and grow. Considering different cell types, beads can be coated with other attachment factors to fit demanding culture conditions.

The matrix selection may lead to different results of cell invasion. Collagen I is the main component of ECM and forms fibrillary networks to withstand stretching. Matrigel is extracted from Engelbreth-Holm-Swarm murine sarcoma and consists of laminin, collagen IV, heparin sulfate proteoglycans, entactin and a few growth factors, which simulates the ECM complex [19]. Here we used growth factor reduced Matrigel so as to decrease the impact of these factors on cell proliferation and invasion. To examine cell invasiveness both of the matrices mentioned above can be used in this method. Importantly, other types of matrices extracted from animal or human tissues can be used as an alternative as long as the matrix can solidify at 37 °C [20]. Moreover, modification of the matrix by adding ECM components enables fine tuning of the conditions in which the cells reside. Our results indicate that the content and concentration of matrix will affect cell performance and therefore results. For appropriate use of this method we recommend to choose or modify the matrix according to the experimental design, and to try different concentrations or compositions if necessary.

In this study we dilute matrix with serum-free medium to generate a determined concentration. On top of the gel culture medium is added to maintain cell growth and prevent gel from drying out. To examine if agents added to the culture medium would influence cell behavior, we compared cell invasion when exposed to 10 or 20% serum. Although a higher serum concentration did not increase the outcome significantly, a positive trend was observed because of enhanced cell proliferation with or without migration. Cell proliferation is inevitable but can be reduced by decreasing the concentration of serum or other growth promoting supplements. Our results indicate that nutrients or treatments in the medium can penetrate into the gel and act on the cells. So, to test different treatments in this 3D invasion assay, growth factors, inhibitors or drugs can be supplemented either in the medium or directly in the gel.

Another interesting finding is that the position of a spheroid in the matrix has an impact on cell moving paths. When spheroids sediment at the bottom of a culture vessel, most cells move along the interface between culture vessel and matrix; while if spheroids are in the middle of two gel layers as a “sandwich”, most cells move between these two gel layers. These observations demonstrate that cells tend to migrate along the path of the least resistance, and researchers need to pay attention to this issue when using this method or similar 3D settings.

Although the microcarrier-based 3D invasion assay has a broad application, the presence of a carrier limits the use to study tumor cell behavior in a spheroid with an anoxic core. Moreover, to study infiltration of tumor cells into a spheroid of normal cells, or to study infiltration of immune cells into a tumor cell spheroid, the assay needs to be extended. A multilayer spheroid can be created over time for this purpose by adjusting the matrix to inhibit migration away from the bead but allow growth. Notably, the described microcarrier-based method cannot be applied to non-adherent cells.

## Conclusions

This study displays a highly reproducible and less time-consuming 3D invasion assay together with practical quantifications and data analysis. Introducing microcarriers to generation of spheroids contributes to uniformity control, short experimental period and the use of a broad range of cell types. We also show time-lapse imaging of cell movement in 3D, which allows visualization of the whole process and advanced analysis. In conclusion, this microcarrier-based 3D invasion assay is a powerful tool to study cell invasion in vitro.

## Methods

### Reagents

Dulbecco’s modified Eagle’s medium (DMEM, D0819, Sigma); Trypsin-EDTA (BE-17-161E, Lonza); Dulbecco’s Phosphate Buffered Saline (PBS, Ca^2+^and Mg^2+^ free, D8537, Sigma-Aldrich); Fetal bovine serum (FBS, F7524, Sigma); Collagen type I, rat tail (08–115; Millipore); Matrigel Growth factor reduced (356,231, Coring); Agar (A1296, Sigma-Aldrich); Sodium bicarbonate (11810–017, Life technologies).

### Imaging System and Climate Control Configuration

**Fig. 6:**
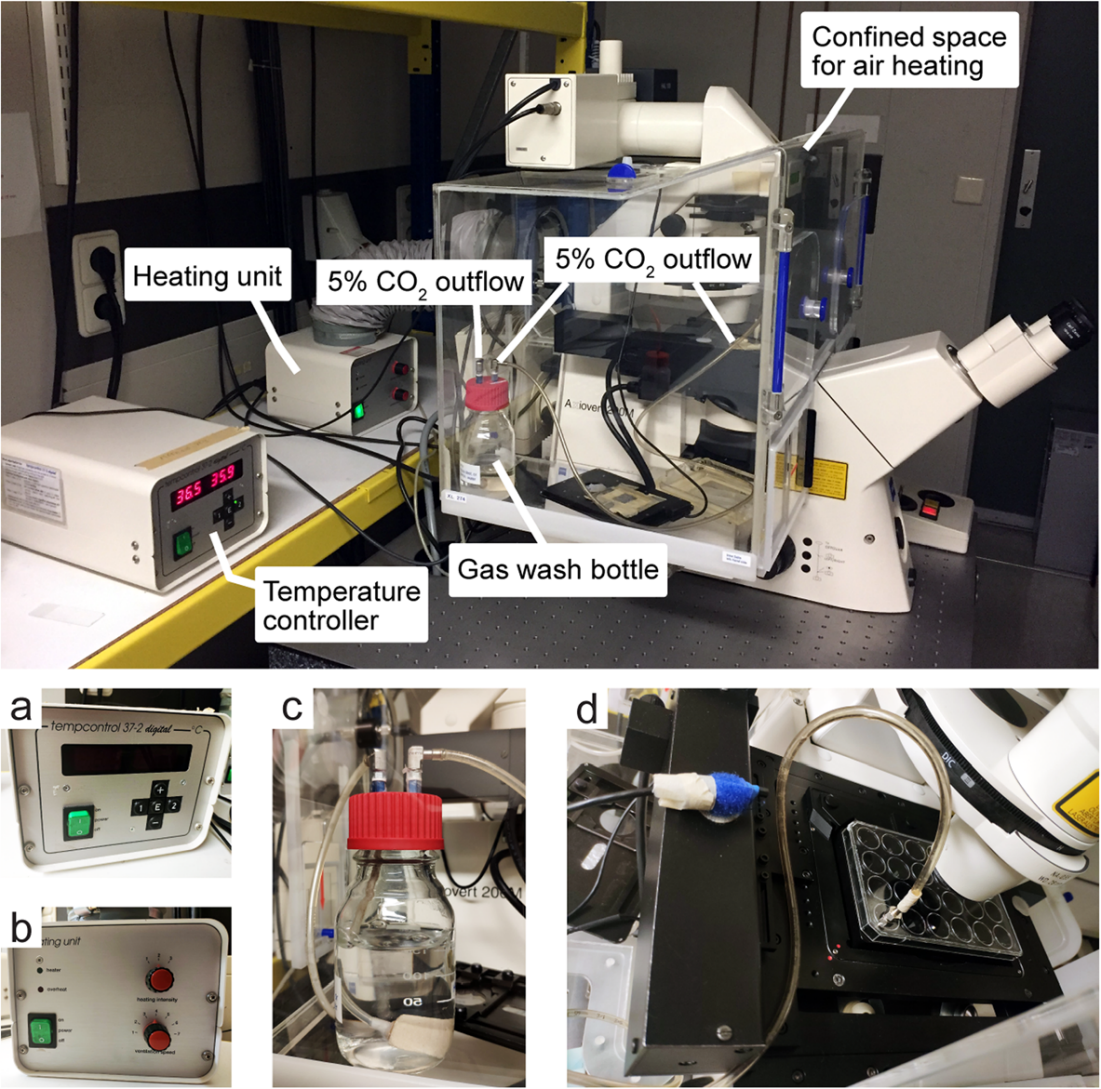
Climate controlled confocal microscopy configuration for time-lapse imaging. **a** Temperature controller. **b** Heating unit. **c** Gas wash bottle. **d** Motorized stage with an experimental plate on top. A tube with humidified airflow containing 5% CO_2_ is connected to the plate

As time-lapse imaging may take hours to days, a screening system, e.g. confocal microscope, integrated with a cell incubation setup is indispensable. Here we show our imaging workspace setup as an example (Fig. 6). A sealed Perspex box was built on the microscope to maintain temperature. The box is heated by a heating unit through a ventilation duct. A sensor in the box is connected to the temperature controller normally set to 37 °C. A 5% CO_2_/air mixture is supplied through a gas wash bottle for humidification, and the flow goes directly to the cell culture plate. Medium evaporation needs to be tested to optimize air flow before experiment. Since cells move in three dimensions in matrices, the microscope with z stacks scanning is recommended for continuous screening with the climate control system. A standard microscope can be used for manual image acquisition as the focus needs to be adjusted over time.

### Preparation of Microcarrier Beads

Cytodex Microcarrier beads (C3275, Sigma-Aldrich) were hydrated in PBS for at least 3 h at room temperature. After beads settlement, discard the supernatant and add fresh Ca^2+^ and Mg^2+^ free PBS to a stock concentration of 50 ml/g. The beads in PBS are sterilized by autoclaving at 120 °C for 20 min and can be stored at 4 °C. Upon use, mix bead suspension in stock thoroughly and pipette 1 ml to a 15 ml Falcon tube. Centrifuge the mixture at 400 g for 5 min and aspirate the supernatant carefully. Re-suspend beads in a volume of 10 ml culture medium to make the final suspension.

### Cell Culture

Human melanoma cell lines (BLM, M14 and Mel57), colorectal cancer cell lines (SW480 and CACO2) and mouse Lewis lung carcinoma (LLC) cells were maintained in Dulbecco’s modified Eagle’s medium (DMEM) supplemented with 10% fetal bovine serum (FBS) under conditions of 5% CO_2_ at 37 °C.

### Preparation of Cell Spheroids with Microcarrier Beads

Cells were suspended in culture medium at a density of 2~5 × 10^5^ cells/ml. Add 1 ml cell suspension and 1 ml bead suspension to a round bottom tube with snap cap (352,059, Corning). Place the tube in a 37 °C incubator with 5% CO_2_ for 6 h and gently shake the tube manually every 2 h to allows cells to evenly distribute on the beads. Manually shaking cannot be replaced by a shaker as most cells will not adhere under continuous shaking. After 6 h of incubation, transfer the mixture (2 ml) to a 6-well plate or a 35 mm petri dish and incubate for 1 to 2 days until most beads are fully covered with cells. Gently clap the culture plate to let spheroids detached for further use. The cell number required to obtain a confluent coverage of beads vary for different cell lines, and should be tested beforehand.

### Embedding Spheroids into Matrix Gel

Spheroid suspension was transferred to a Falcon tube and left for 5 min allowing spheroids to settle. Aspirate all culture medium carefully and add the same amount (2 ml) of DMEM to re-suspend spheroids. Prepare a certain concentration of matrix with collagen (option A), Matrigel (option B) or agar (option C). The recommended concentration of collagen type I is 1.4–2.3 mg/ml according to the quantity of collagen I in human fresh tissue [21]. For Matrigel, the concentration that forms a solid gel and allows cells to invade properly in 2 to 3 days (e.g. 4–5 mg/ml) should be determined in pilot assays before further experimentation, as it may vary between companies and batches. Here we show the volume of reagents for duplicates preparation in a 24-well format.

(A) Collagen gel formulation for cell invasion
  (i) Keep collagen on ice. Pre-chill pipette tips and Eppendorf tubes used for matrix preparation.
  (ii) Mix 340 μl DMEM and 27 μl 7.5% (w/v) NaHCO_3_ in a sterile Eppendorf tube.
  (iii) Add 100 μl spheroid suspension to the Eppendorf tube. Slowly add 533 μl collagen (3 mg/ml) and gently pipette up and down to mix well. The final concentration of collagen is 1.6 mg/ml. Dispense 400 μl mixture in each well without air bubbles and incubate the plate at 37 °C for at least 30 min until a solid gel formed.
  (iv) Add 500 μl warm (37 °C) culture medium carefully along the side wall onto the gel. To investigate treatment effects, agents can be mixed in the culture medium before adding to the gel.
(B) Matrigel formulation for cell invasion
  (i) Keep Matrigel on ice. Pre-chill pipette tips and Eppendorf tubes used for matrix preparation.
  (ii) Add 440 μl DMEM and 100 μl spheroid suspension to a sterile Eppendorf tube.
  (iii) Slowly add 460 μl Matrigel GFR (10.9 mg/ml) and gently pipette up and down to mix well. The final concentration of Matrigel is 5 mg/ml. Dispense 400 μl mixture in each well without air bubbles and incubate the plate at 37 °C for at least 30 min until a solid gel formed.
  (iv) Add 500 μl warm (37 °C) culture medium carefully along the side wall onto the gel. To investigate treatment effects, agents can be mixed in the culture medium before adding to the gel.
(C) Agar formulation for cell invasion
  (i) Sterilize 0.6% (w/v) agar by autoclaving at 120 °C for 20 min and store at 4 °C. Before use agar should be completely boiled in a microwave and mixed well. Keep agar in a 42 °C water bath to prevent solidification.
  (ii) Mix 375 μl DMEM and 25 μl 7.5% NaHCO_3_ in a sterile Eppendorf tube.
  (iii) Add 100 μl spheroids suspension to the Eppendorf tube. Slowly add 500 μl 0.6% agar and gently pipette up and down to mix well. The final concentration of agar is 0.3%. Dispense 400 μl of the mixture in each well without air bubbles and incubate the plate at room temperature for 20–30 min until a solid gel formed.
  (iv) Add 500 μl warm (37 °C) culture medium carefully along the side wall onto the gel. To investigate treatment effects, agents can be mixed in the culture medium before adding to the gel.

### Imaging Cell Invasion in Matrix

Cell invasion can be monitored by time-lapse microscopy (option A) for several days. It requires a climate control system to keep cells alive during imaging. Here we use a confocal microscope installed with a cell culture box. A sealed Perspex box is built on the microscope to maintain temperature. Assemble the heating unit to warm the air inside the Perspex box and the motorized stage where culture plate is placed. A 5% CO_2_/air mixture is supplied through a heated gas wash bottle for humidification, and it goes directly to the cell culture plate or chamber on the motorized stage (Fig. 6). The flow rate needs to be low to prevent evaporation of medium in the plate, and it can be adjusted based on the frequency of air bubbles in the gas wash bottle. In the absence of a climate controlled configuration, it is also possible to image cell dispersion and invasion manually (option B). Image acquisition in bright field or fluorescence can be done in this setting, and several time points were recorded.

(A) Time-lapse imaging
  (i) Switch heating unit on and set it to 37 °C before imaging to ensure the heating is stable.
  (ii) Place the experimental plate or cell chamber on the stage of the confocal microscope and let temperature, CO_2_ and humidity stabilize.
  (iii) Turn on and configure the confocal imaging software to appropriate settings (e.g. lasers, channels, scan parameters). Apply the same configuration when repeating experiments.
  (iv) Browse spheroid distribution in matrix with a 10× 0.3NA Plan-Neofluar objective lens. Choose a spheroid which is fully covered with cells and far enough from other spheroids. Adjust the position to center the spheroid of interest in the middle of the image and save this position in the location list. Repeat this step to find other spheroids and save their coordinates.
  (v) Set z stack interval and range. The interval is determined by the pinhole. The range is usually set to ~ 200 μm and can be adjusted for different cells.
  (vi) Determine the time interval and repetitions which vary depending on cell invasion ability. Usually we set the time interval to 30 min and the duration to 2–3 days.
  (vii) Start imaging and check if the setup runs well during imaging acquisition. In particular check above mentioned environmental settings and whether evaporation of medium occurs.
(B) Imaging cell dispersion manually
  (i) Place the multi-well plate or culture chamber on the stage of a standard microscope.
  (ii) Turn on the imaging software connected to the microscope and set up for image acquisition in bright-field or fluorescence. The software needs to display x and y coordinates.
  (iii) Make a mark with a pen on the upper left corner of the plate and set this mark to 0 position manually. Browse spheroids distribution in matrix under a 10× objective lens. Choose spheroids which are fully covered with cells and far enough from other spheroids. Adjust the position to center the spheroid of interest in the middle of the image, save this position in the location list and take a picture as T = 0. Repeat this for other spheroids of interest. After photographing all the selected spheroids, put the plate back in the incubator.
  (iv) Pictures of the same spheroids can be taken every 12 or 24 h until cells spread out of frame or at a desired end point of this experiment. At every time point, reset the mark at the 0 position before taking pictures to avoid shifting of position.

### Quantification of Migratory Parameters and Data Analysis

**Fig. 7:**
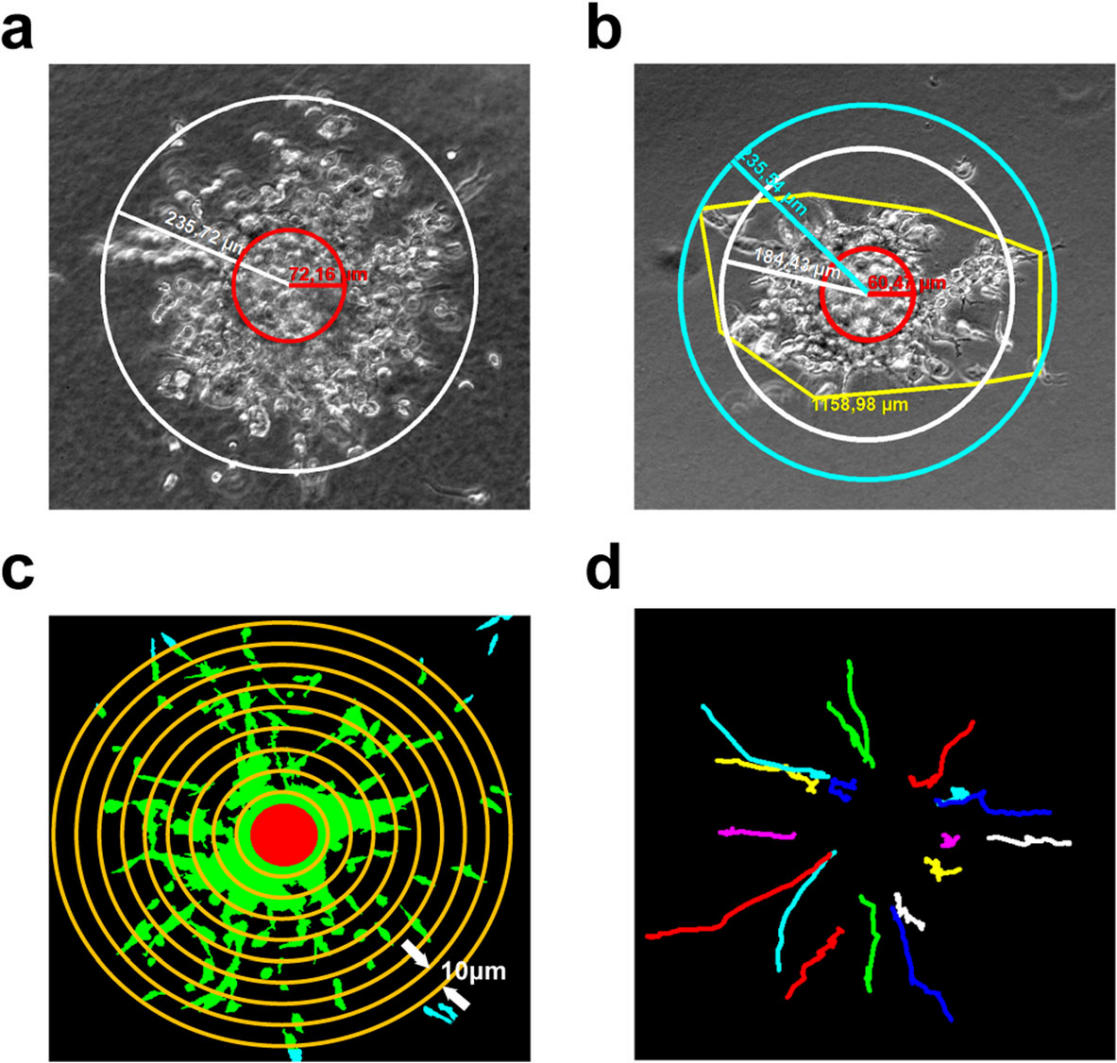
Quantification of migratory parameters. **a** Maximum migrating distance measured when cells evenly distributed in all directions. White circle, cell migration front. Red circle, size of bead. **b** Average of maximum migrating distance applied when cells showed uneven distribution in a shape of a polygon rather than a sphere. Yellow curve, cell migration front. White circle, calculated average of maximum distance. Light blue circle, maximum of cell migration front. Red circle, size of bead. **c** Schematic diagram to show the principle of computing migration area on the basis of the distance to the core. Cells are selected and filled with green. Light blue represents cells out of range. Red core is where the bead resides. Yellow concentric circles with radius difference of 10 μm are drawn to measure the migration area of increasing distance to beads. In this schematic the yellow circles do not have a radius difference of exact 10 μm but were only drawn to illustrate this quantification method. **d** Cell trajectories in collagen I between 55 to 70 h, tracked manually. Panels a-b show representative images of M14 cells, and panels c-d show examples of quantification on LLC cell images

Several methods can be performed to quantify migratory parameters under different conditions. The maximum migrating distance (option A) or the average of maximum migrating distance (option B) is applied when cells migrate cohesively and very few cells move far away from the cell cluster (Fig. 7a,b). Here we use the AxioVision image analysis module as an example to measure these parameters which can alternatively be done in Fiji [22] or similar software. Some cell lines move individually or follow a path created by front cells, thus show spotted or radiating/sprouting dispersion respectively. In this case a migration index (option C) can be applied to determine cell invasion characteristics. The migration index is defined as the sum of all migrating cells multiplied with the distance from the bead. If time-lapse imaging is conducted, moving trajectories of individual cells can be tracked manually or with tracking software from which migration distances and velocity are calculated (option D).

(A) Measuring maximum migrating distance
  (i) Open file at a time point in AxioVision. Under “Measure” menu select the “Circle” tool.
  (ii) Draw a circle matching the bead to measure size of bead (Fig. 7a, red circle). From the center draw another circle involving all migrating cells (Fig. 7a, white circle).
  (iii) Calculate maximum migrating distance at this time point. Max migrating distance (μm) = radius of migrating front circle – radius of bead circle
(B) Measuring average of maximum migrating distance
  (i) Open file at a time point in AxioVision.
  (ii) Under “Measure” menu select the “Circle” tool. Draw a circle matching the bead to measure size of bead (Fig. 7b, red circle).
  (iii) Under “Measure” menu select the “Curve” tool. Draw a curve along the migrating front to generate a convex polygon (Fig. 7b, yellow curve) to measure the perimeter. Only the perimeter of convex polygon can be used to calculate the radius with this formula [23]. A concave polygon extends the perimeter which causes incorrect result.
  (iv) Calculate average of maximum migrating distance at this time point (Fig. 7b, white circle). Avg. max migrating distance (μm) = (perimeter of the polygon/2π) – radius of bead circle
(C) Computing migration index
  (i) Open file with z stack at a selected time point in Fiji.
  (ii) Find the contours of the bead in the spheroid by browsing through the z stack and draw a circle (Circle0) matching the biggest bead diameter. Record this instruction in the macro recorder.
  (iii) Make a z projection of the original file. Set threshold to include all cells. Recreate Circle0 by running the recorded macro. Measure the area of the circle (Area0).
  (iv) Draw Circle1 with the same center as Circle0 and radius 10 μm larger than Circle0. Area1 = area of Circle1 - Area0. Each time draw a circle 10 μm larger than the previous one and measure the area until the circle reaches the edge of image (Fig. 7c). The whole automated image processing macros can be found in Additional file 1.
  (v) Export results to Excel. Calculate the increasing area of each circle. Area(*i)* = area of Circle(*i)* – area of Circle*(i-1)* where *i* = 1, 2, 3, … max number of circles. A graph can be drawn to display distribution of cells around the bead at this time point, in which x axis represents distance to bead and y axis represents migration area (Fig. 4b).
  (vi) If we assume every cell has the same size, then the area is proportional to cell numbers. The migration index can be calculated using the equation:
    $$ Migration\ index=\sum \limits_{i=1}^n10\times i\times \mathrm{Area}(i) $$

where n is the maximum number of circles. This formula is adapted from Jozaki, K. et al. [24].

(D) Cell trajectory and velocity
  (i) Open the time-lapse sequence of each selected position in Fiji.
  (ii) Make a z projection and adjust brightness and color to make cells easily recognized.
  (iii) Use “Manual tracking” plug-in to track individual cells (Fig. 7d). Results will show distance and velocity between every two slices. Export results in Excel and calculate the migration distance and velocity. Other automated tracking methods are available for analysis [25, 26].

## Supplementary information

**Additional file 1.** Homemade macros to quantify migrating cell areas of each distance range in Fiji.

## Data Availability

All data generated or analyzed during this study are included in this published article and its supplementary information files.

## Abbreviations

2D: Two dimensions
3D: Three dimensions
DMEM: Dulbecco’s modified Eagle’s medium
ECM: Extracellular matrix
FBS: Fetal bovine serum
GFR: Growth factor reduced
LLC: Lewis lung carcinoma
NS: Not significant
PBS: Phosphate buffered saline

## References

[1] Chambers AF, Groom AC, MacDonald IC (2002). Dissemination and growth of cancer cells in metastatic sites. Nat Rev Cancer.

[2] Friedl P, Wolf K (2003). Tumour-cell invasion and migration: diversity and escape mechanisms. Nat Rev Cancer.

[3] Sahai E (2007). Illuminating the metastatic process. Nat Rev Cancer.

[4] Christiansen JJ, Rajasekaran AK (2006). Reassessing epithelial to mesenchymal transition as a prerequisite for carcinoma invasion and metastasis. Cancer Res.

[5] Friedl P, Locker J, Sahai E, Segall JE (2012). Classifying collective cancer cell invasion. Nat Cell Biol.

[6] Albini A, Benelli R (2007). The chemoinvasion assay: a method to assess tumor and endothelial cell invasion and its modulation. Nat Protoc.

[7] Liu H, Zhu M, Li Z, Wang Y, Xing R, Lu Y (2017). Depletion of p42.3 gene inhibits proliferation and invasion in melanoma cells. J Cancer Res Clin Oncol.

[8] Benien P, Swami A (2014). 3D tumor models: history, advances and future perspectives. Future Oncol.

[9] Nath S, Devi GR (2016). Three-dimensional culture systems in cancer research: focus on tumor spheroid model. Pharmacol Ther.

[10] Vinci M, Gowan S, Boxall F, Patterson L, Zimmermann M, Court W (2012). Advances in establishment and analysis of three-dimensional tumor spheroid-based functional assays for target validation and drug evaluation. BMC Biol.

[11] Lee JM, Mhawech-Fauceglia P, Lee N, Parsanian LC, Lin YG, Gayther SA (2013). A three-dimensional microenvironment alters protein expression and chemosensitivity of epithelial ovarian cancer cells in vitro. Lab Investig.

[12] Dolznig H, Rupp C, Puri C, Haslinger C, Schweifer N, Wieser E (2011). Modeling colon adenocarcinomas in vitro a 3D co-culture system induces cancer-relevant pathways upon tumor cell and stromal fibroblast interaction. Am J Pathol.

[13] Johns RA, Tichotsky A, Muro M, Spaeth JP, Le Cras TD, Rengasamy A (1995). Halothane and isoflurane inhibit endothelium-derived relaxing factor-dependent cyclic guanosine monophosphate accumulation in endothelial cell-vascular smooth muscle co-cultures independent of an effect on guanylyl cyclase activation. Anesthesiology..

[14] Bakker ER, Das AM, Helvensteijn W, Franken PF, Swagemakers S, van der Valk MA (2013). Wnt5a promotes human colon cancer cell migration and invasion but does not augment intestinal tumorigenesis in Apc1638N mice. Carcinogenesis..

[15] Janmaat VT, Liu H, da Silva RA, Wisse PHA, Spaander MCW, Ten Hagen TLM, et al. HOXA9 mediates and marks premalignant compartment size expansion in colonic adenomas. Carcinogenesis. 2019:bgz038. 10.1093/carcin/bgz038. 10.1093/carcin/bgz038 31099823

[16] Dietrich F, Lelkes PI (2006). Fine-tuning of a three-dimensional microcarrier-based angiogenesis assay for the analysis of endothelial-mesenchymal cell co-cultures in fibrin and collagen gels. Angiogenesis..

[17] Kniazeva E, Putnam AJ (2009). Endothelial cell traction and ECM density influence both capillary morphogenesis and maintenance in 3-D. Am J Phys Cell Phys.

[18] Juliar BA, Keating MT, Kong YP, Botvinick EL, Putnam AJ (2018). Sprouting angiogenesis induces significant mechanical heterogeneities and ECM stiffening across length scales in fibrin hydrogels. Biomaterials..

[19] Kleinman HK, Martin GR (2005). Matrigel: basement membrane matrix with biological activity. Semin Cancer Biol.

[20] Kaukonen R, Jacquemet G, Hamidi H, Ivaska J (2017). Cell-derived matrices for studying cell proliferation and directional migration in a complex 3D microenvironment. Nat Protoc.

[21] Rojkind M, Giambrone MA, Biempica L (1979). Collagen types in normal and cirrhotic liver. Gastroenterology..

[22] Schindelin J, Arganda-Carreras I, Frise E, Kaynig V, Longair M, Pietzsch T (2012). Fiji: an open-source platform for biological-image analysis. Nat Methods.

[23] Merkus HG (2009). Particle size measurements: fundamentals, practice, quality: Springer Science & Business Media, Dordrecht, the Netherlands; 2009 7 Jannuary.

[24] Jozaki K, Marucha PT, Despins AW, Kreutzer DL (1990). An in vitro model of cell migration: evaluation of vascular endothelial cell migration. Anal Biochem.

[25] Tokunaga T, Hirose O, Kawaguchi S, Toyoshima Y, Teramoto T, Ikebata H (2014). Automated detection and tracking of many cells by using 4D live-cell imaging data. Bioinformatics..

[26] Dzyubachyk O, van Cappellen WA, Essers J, Niessen WJ, Meijering E (2010). Advanced level-set-based cell tracking in time-lapse fluorescence microscopy. IEEE Trans Med Imaging.

